# Characterization of *blaNDM-1* and *blaOXA-23*-Type Carbapenemases in *Acinetobacter baumannii* Isolated from Post-Surgical Patients

**DOI:** 10.3390/antibiotics15080722

**Published:** 2026-07-24

**Authors:** Sana Gul, Nawab Ali, Muhammad Qasim, Maali Alahmad, Muhammad Saeed Khan, Zull E. Nourain, Sadir Zaman, Yar Muhammad, Waheed Ullah

**Affiliations:** 1 Department of Microbiology, Kohat University of Science and Technology, Kohat 26000, Khyber Pakhtunkhwa, Pakistan; sanagulgulalay@gmail.com (S.G.); qasim89@gmail.com (M.Q.); saeedhist@gmail.com (M.S.K.); muhammadzullenourain@gmail.com (Z.E.N.); sadirzaman33@gmail.com (S.Z.); 2 College of Engineering and Energy, Abdullah Al Salem University, Khaldiya Campus, Kuwait City 72300, Kuwait; 3 College of Integrative Studies, Abdullah Al Salem University, Kuwait City 72300, Kuwait; maali.alahmad@aasu.edu.kw; 4 Department of Biotechnology, University of Science and Technology, Bannu 28100, Khyber Pakhtunkhwa, Pakistan; janbaznurar@yahoo.com

**Keywords:** *Acinetobacter baumannii*, surgical site infections, carbapenem resistance, *blaOXA-23*, *blaNDM-1*, multidrug resistance, Pakistan

## Abstract

**Background:** *Acinetobacter baumannii* is an important opportunistic pathogen responsible for healthcare-associated infections, particularly among critically ill, intensive care unit (ICU), and post-surgical patients. The emergence of carbapenem-resistant *A. baumannii* (CRAB) has become a major therapeutic challenge worldwide because of its multidrug-resistant nature and limited treatment options. Despite the increasing prevalence of CRAB in Pakistan, information regarding the molecular characterization of carbapenem resistance genes among isolates recovered from surgical site infections (SSIs) remains limited. **Methods:** A cross-sectional study was conducted from November 2023 to February 2024 at Khalifa Gul Nawaz Hospital, Bannu, Khyber Pakhtunkhwa, Pakistan. A total of (n = 118) surgical wound specimens were collected from patients with clinically diagnosed SSIs. *A. baumannii* isolates were identified using standard biochemical tests and confirmed by 16S rRNA gene sequencing. In vitro antimicrobial susceptibility was determined by Kirby–Bauer disk diffusion according to CLSI 2024 guidelines. In addition, the concentration-dependent inhibition-zone response of imipenem and meropenem against carbapenem-resistant isolates was evaluated using an agar well diffusion assay. Molecular detection of *blaNDM-1* and *blaOXA-23* was performed by polymerase chain reaction (PCR), and representative amplicons were subjected to Sanger sequencing for further analysis. **Results:** In the current study, the total number of (n = 118) wound specimens from healthcare-associated patients were processed, in which *A. baumannii*-positive isolates (n = 23, 19.5%) were documented. Its prevalence was higher in males (69.6%) compared to females, and was strongly associated (78.2%) with elderly patients (aged 51–68 years). In vitro susceptibility testing revealed that 91.3% of isolates harbored multidrug-resistant (MDR) attributes. Antimicrobial susceptibility profiling revealed high levels of multidrug resistance, with 82.6% of isolates resistant to imipenem and meropenem, as well as 100% resistance to aztreonam and gentamicin. Colistin showed the highest activity, with 87.0% of isolates remaining susceptible. In the agar well diffusion assay, measurable inhibition of the carbapenem-resistant isolates by imipenem and meropenem occurred only at higher concentrations. Molecular screening of resistance genes identified *blaOXA-23* in 60.9% and *blaNDM-1* in 30.4% of isolates. Co-expression of both genes was detected in some (n = 4) isolates. Among the selected MDR isolates included in the gene resistance analysis, imipenem and meropenem resistance were recorded in 9/14 *bla*OXA-23-positive isolates and 5/7 *bla*NDM-1-positive isolates. **Conclusions:** This study substantiates an escalated incidence of carbapenem-resistant, MDR *A. baumannii* in post-surgical infections. *blaOXA-23* were documented as the predominant carbapenemase gene, with co-expression of *blaNDM-1* in a substantial proportion of isolates. These findings provide an important phenotypic and molecular characterization of selected carbapenemase genes in healthcare-associated infections, highlighting the exigency for strengthened antimicrobial stewardship and infection control strategies in the hospitals of the region.

## 1. Introduction

*Acinetobacter baumannii* is an opportunistic Gram-negative coccobacillus and one of the most important causes of healthcare-associated infections (HAIs), particularly among critically ill, immuno-compromised, intensive care unit (ICU), and post-surgical patients [1]. It is responsible for a wide range of infections, including ventilator-associated pneumonia, bloodstream infections, urinary tract infections, wound infections, and healthcare-associated infections [2]. The strong potential of *A. baumannii* to survive under adverse environmental conditions, persist on hospital surfaces, and rapidly acquire antimicrobial resistance has facilitated its global dissemination, making it a major challenge for modern healthcare systems [3].

Carbapenems, particularly imipenem and meropenem, have been previously considered an effective antibiotic against multidrug-resistant Gram-negative bacterial pathogens. However, increasing carbapenem resistance in *A. baumannii* has become a major therapeutic concern worldwide [4]. Resistance is primarily mediated by carbapenem-hydrolyzing enzymes, especially class D oxacillinases and class B metallo-β-lactamases, although additional mechanisms such as efflux pump overexpression and porin alterations also have significance in importing resistance [5]. Among the various carbapenemase genes identified in *A. baumannii*, *blaOXA-23* and *blaNDM-1* are considered the most clinically significant because of their widespread distribution within the community and strong association with carbapenem resistance [6]. The *blaOXA-23* gene is the predominant acquired oxacillinase reported in *A. baumannii*, whereas *blaNDM-1* has emerged as an important metallo-β-lactamase contributing to high-level resistance in many countries, including Pakistan [7]. In Pakistan, the prevalence of carbapenem-resistant *A. baumannii* has increased considerably during the past decade, particularly in tertiary-care hospitals [8]. Although several studies have reported antimicrobial resistance patterns among clinical isolates, molecular characterization of carbapenem resistance genes in isolates recovered specifically from surgical site infections remains limited [9]. Despite the increasing burden of carbapenem-resistant *A. baumannii* in Pakistan, limited information is available regarding the distribution of *blaOXA-23* and *blaNDM-1* among isolates recovered from surgical site infections in Khyber Pakhtunkhwa. Moreover, few local studies have combined phenotypic antimicrobial susceptibility testing with molecular detection of these clinically important carbapenemase genes. Therefore, the present study aimed to determine the occurrence of *A. baumannii* among surgical site infections, evaluate the antimicrobial susceptibility profile of the recovered isolates, and characterize the presence of *blaOXA-23* and *blaNDM-1* carbapenemase genes.

## 2. Results

### 2.1. Isolation and Identification of A. baumannii

A total of (n = 118) surgical wound specimens were collected from patients with clinically diagnosed SSIs during the study period (November 2023 to February 2024). *A. baumannii* was successfully isolated from 23 specimens, yielding an overall isolation rate of 19.5% (23/118). All isolates were preliminarily identified based on their morphological and biochemical properties. Among the (n = 23) *A. baumannii*-positive cases, (n = 16, 69.6%) were male and (n = 7, 30.4%) were female. The age-wise distribution revealed that one isolate (4.5%) belonged to the age group of 10–22 years, six isolates (26.0%) to 24–50 years, and 16 isolates (69.5%) to 51–68 years, as shown in Table 1. *A. baumannii* were strongly associated with diabetic foot infections (47%, n = 11), followed by post-mesh hernia infections (34%, n = 8) and post-appendectomy wound infections (17%, n = 4).

### 2.2. In Vitro Antimicrobial Susceptibility Profiles of A. baumannii Isolates

In vitro antimicrobial susceptibility testing revealed a high incidence of MDR *A. baumannii* in SSIs. Overall, 21 of 23 isolates (91.3%) met the criteria for MDR, defined as resistance to at least one agent from three or more antibiotic classes. Carbapenem resistance was detected in 19 isolates (82.6%) for imipenem and meropenem, complete resistance (100%) was observed with aztreonam and gentamicin, and resistance to fluoroquinolones (levofloxacin, ciprofloxacin, ofloxacin) was detected in 21 isolates (91.3%) each. Among cephalosporins, the highest resistance (n = 18, 78.3%) was observed against ceftazidime and cefepime. Among all the tested antibiotics, colistin was the most effective agent against *A. baumannii*, followed by polymyxin B. Colistin resistance was detected in nine isolates (39.1%), representing a clinically concerning finding given its last-resort status in the treatment of CRAB infections, all of which is shown in Table 2.

### 2.3. Concentration-Dependent Agar Well Diffusion Assay of Imipenem and Meropenem

Agar well diffusion testing demonstrated a clear concentration-dependent increase in the inhibition-zone diameter for both imipenem and meropenem against (n = 21) MDR *A. baumannii* isolates (Table 3). For imipenem, no inhibition zone was observed at 10 or 13 µg/mL. A measurable zone first appeared at 15 µg/mL (8 mm), and increased progressively with concentrations of 17 µg/mL (11 mm), 19 µg/mL (14 mm), 21 µg/mL (17 mm), 23 µg/mL (20 mm), and 25 µg/mL (23 mm), reaching a maximum of 25 mm at 27 µg/mL. For meropenem, a small zone was detectable from the lowest concentration tested (7 mm at 10 µg/mL) and increased steadily to a maximum of 25 mm at 27 µg/mL.

### 2.4. Molecular Characterization and Phylogenetic Analysis of A. baumannii

**Figure 1:**
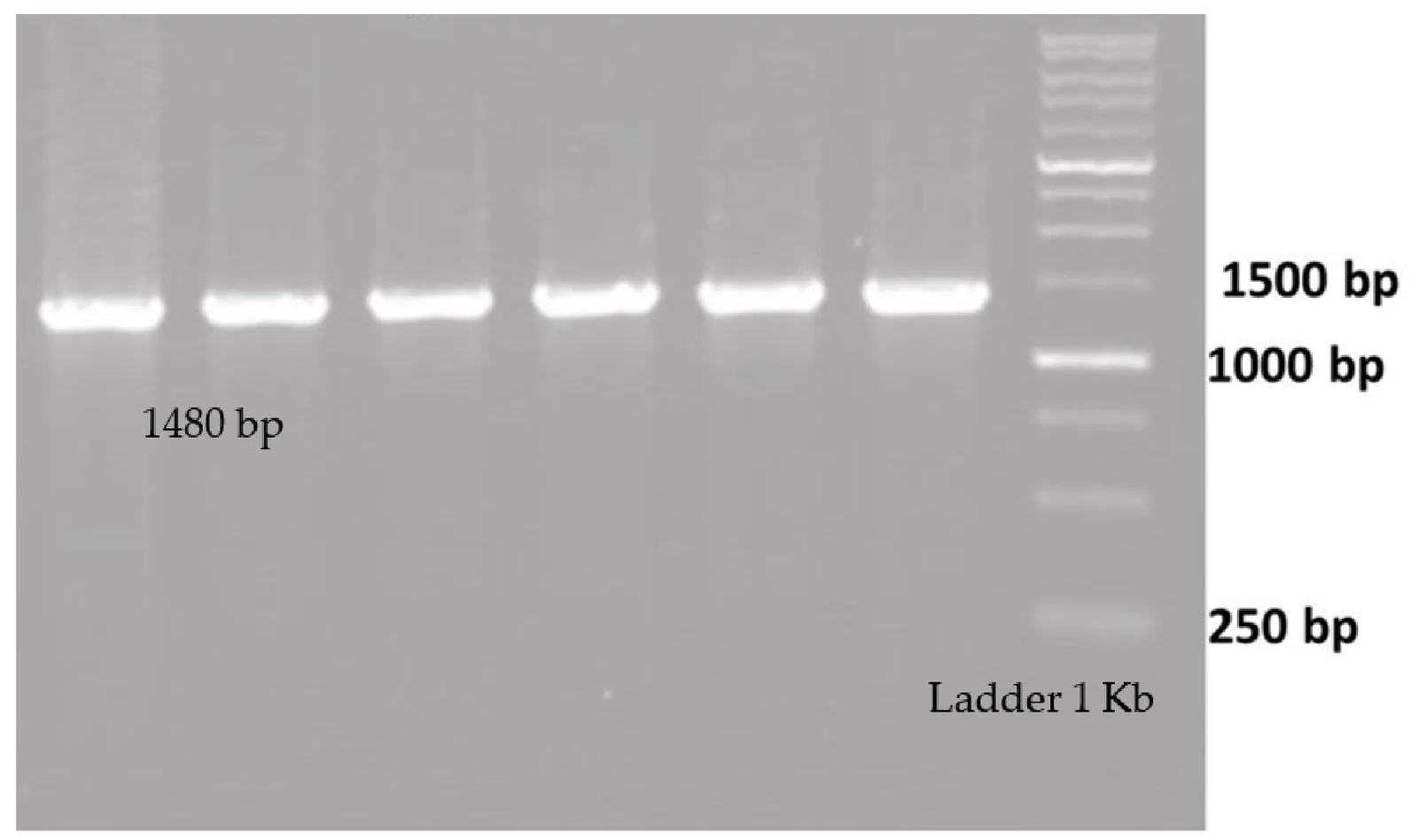
**Agarose gel electrophoresis of PCR-amplified 16S rRNA gene fragments from representative *Acinetobacter baumannii* isolates.** Gel bands show a 1 kb DNA ladder and positive PCR products. A single amplicon of approximately 1480 bp was detected in each isolate, confirming successful amplification of the 16S rRNA gene for molecular identification.

**Figure 2:**
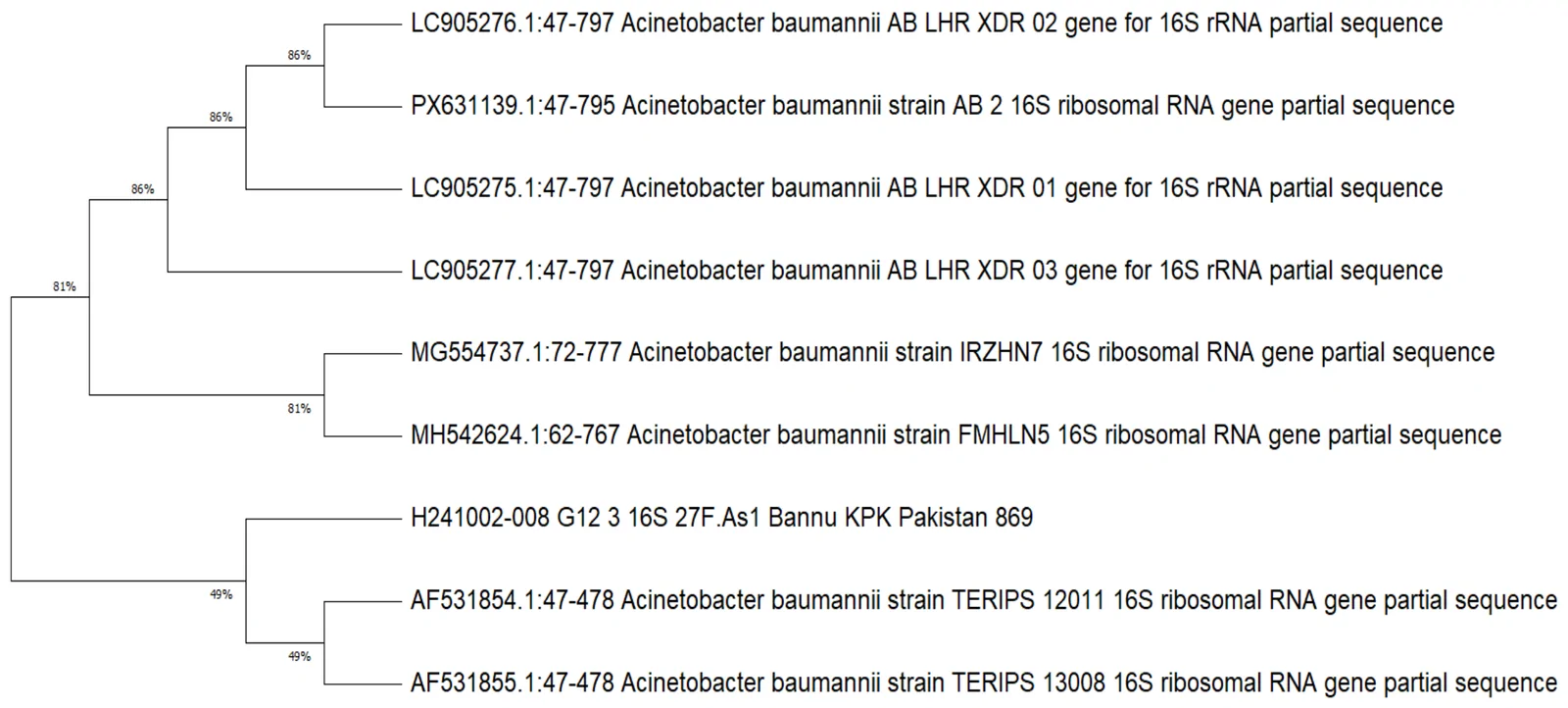
Neighbor-joining phylogenetic tree constructed based on the partial 16S rRNA gene sequences of *Acinetobacter baumannii* isolates recovered in the present study and closely related reference sequences retrieved from the NCBI GenBank database. Bootstrap values based on 1000 replications are indicated at each node. Scale bar represents nucleotide substitutions per site. The study isolate is denoted as AS1.

PCR amplification of the 16S rRNA gene yielded a 1480 bp product in all 23 *A. baumannii* isolates as shown in (Figure 1). BLASTn analysis of the representative sequenced products demonstrated 98–100% nucleotide identity with reference *A. baumannii* sequences deposited in the NCBI GenBank database, confirming the molecular identity of all isolates (Figure 2). Sequence-based comparison showed that the study isolates clustered closely with geographically diverse reference strains of *A. baumannii*, consistent with the conserved nature of the 16S rRNA gene- and species-level identity of the isolates.

### 2.5. PCR-Based Detection of Carbapenem Resistance Genes

**Figure 3:**
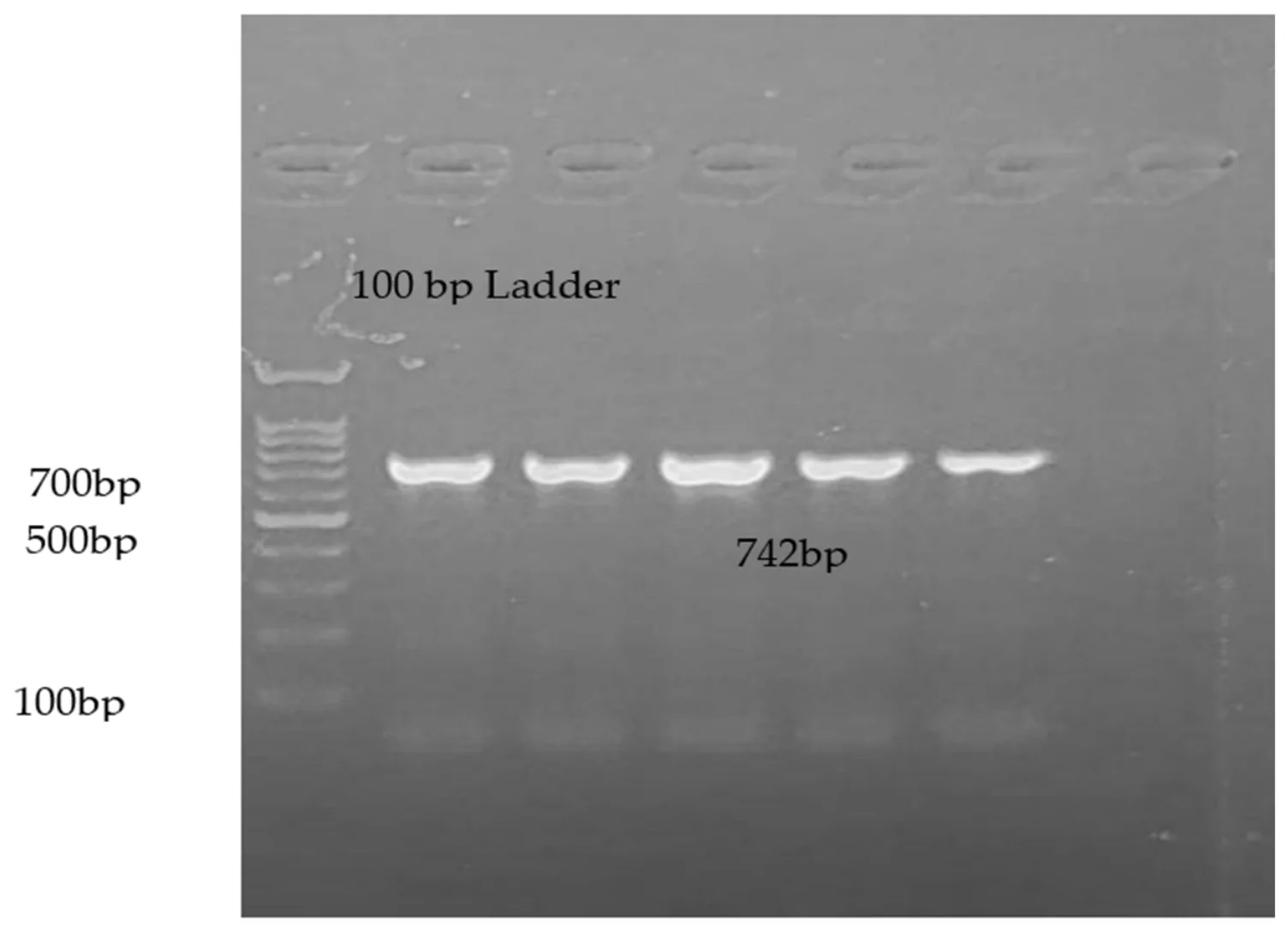
Agarose gel electrophoresis of PCR-amplified *blaOXA-23* gene fragments from *Acinetobacter baumannii* isolates. Gel bands show a 100 bp DNA ladder and positive PCR products. A single amplicon of approximately 742 bp was detected in each isolate.

**Figure 4:**
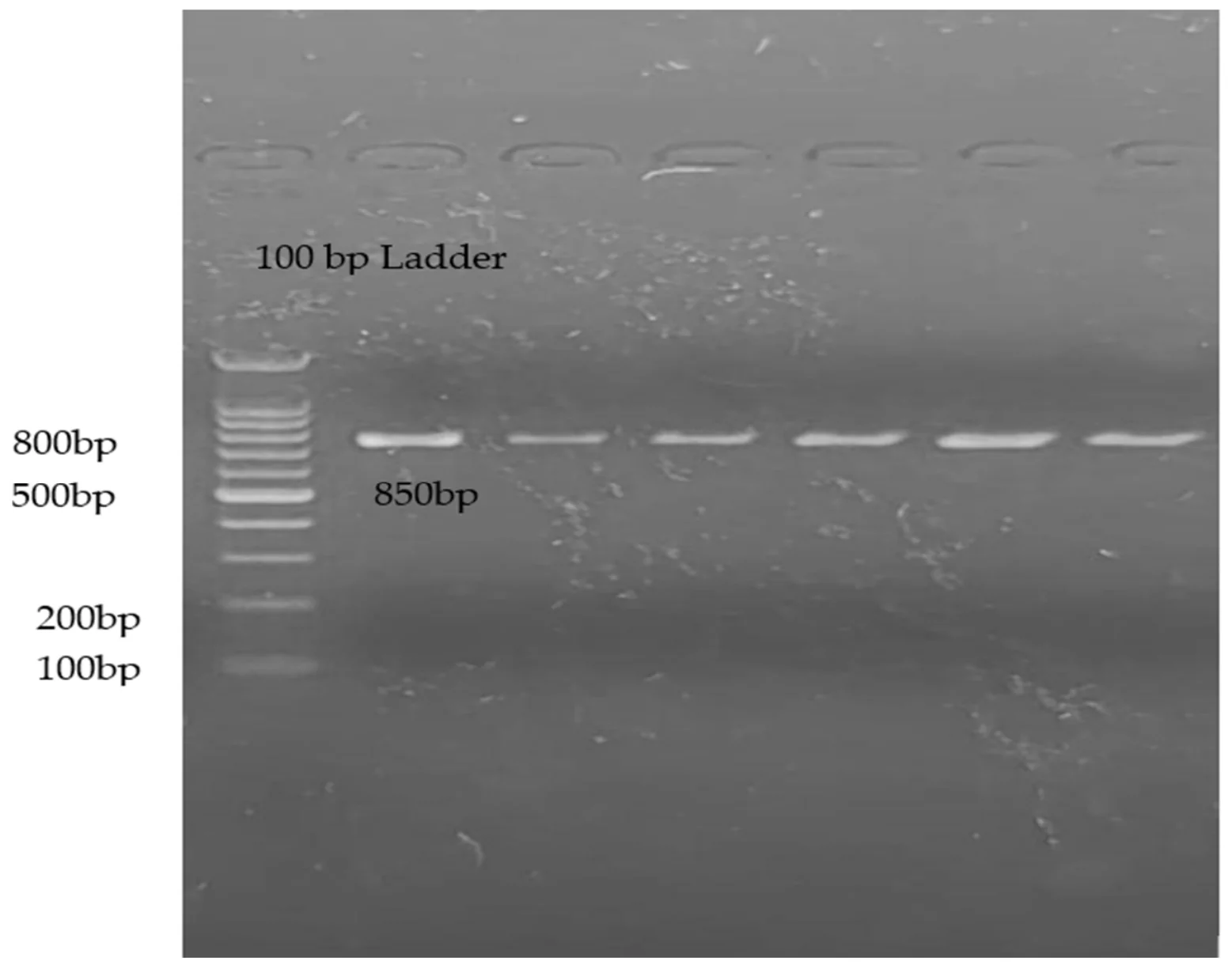
Agarose gel electrophoresis of PCR-amplified *blaNDM-1* gene fragments from *Acinetobacter baumannii* isolates. Gel bands show a 100 bp DNA ladder and positive PCR products. A single amplicon of approximately 850 bp was detected in each isolate.

PCR screening identified carbapenemase-encoding genes in a substantial proportion of A. baumannii isolates. *blaOXA-23* was detected in 14 isolates (60.9%), while *blaNDM-1* was identified in seven isolates (30.4%). Co-detection of both genes was observed in four isolates (17.4%) and is summarized in Table 4. Agarose gel electrophoresis confirmed the amplification of expected product sizes of 742 bp for *blaOXA-23* and 850 bp for *blaNDM-1* (Figure 3 and Figure 4). Among *bla*OXA-23-positive isolates, resistance to imipenem and meropenem was recorded in 9/14, while in *bla*NDM-1-positive isolates, resistance to imipenem and meropenem was 5/7. These findings indicate that resistance was frequent among both *bla*OXA-23- and *bla*NDM-1-positive isolates, as shown in Table 4.

### 2.6. Sequence Analysis of blaOXA-23 and blaNDM-1

**Figure 5:**
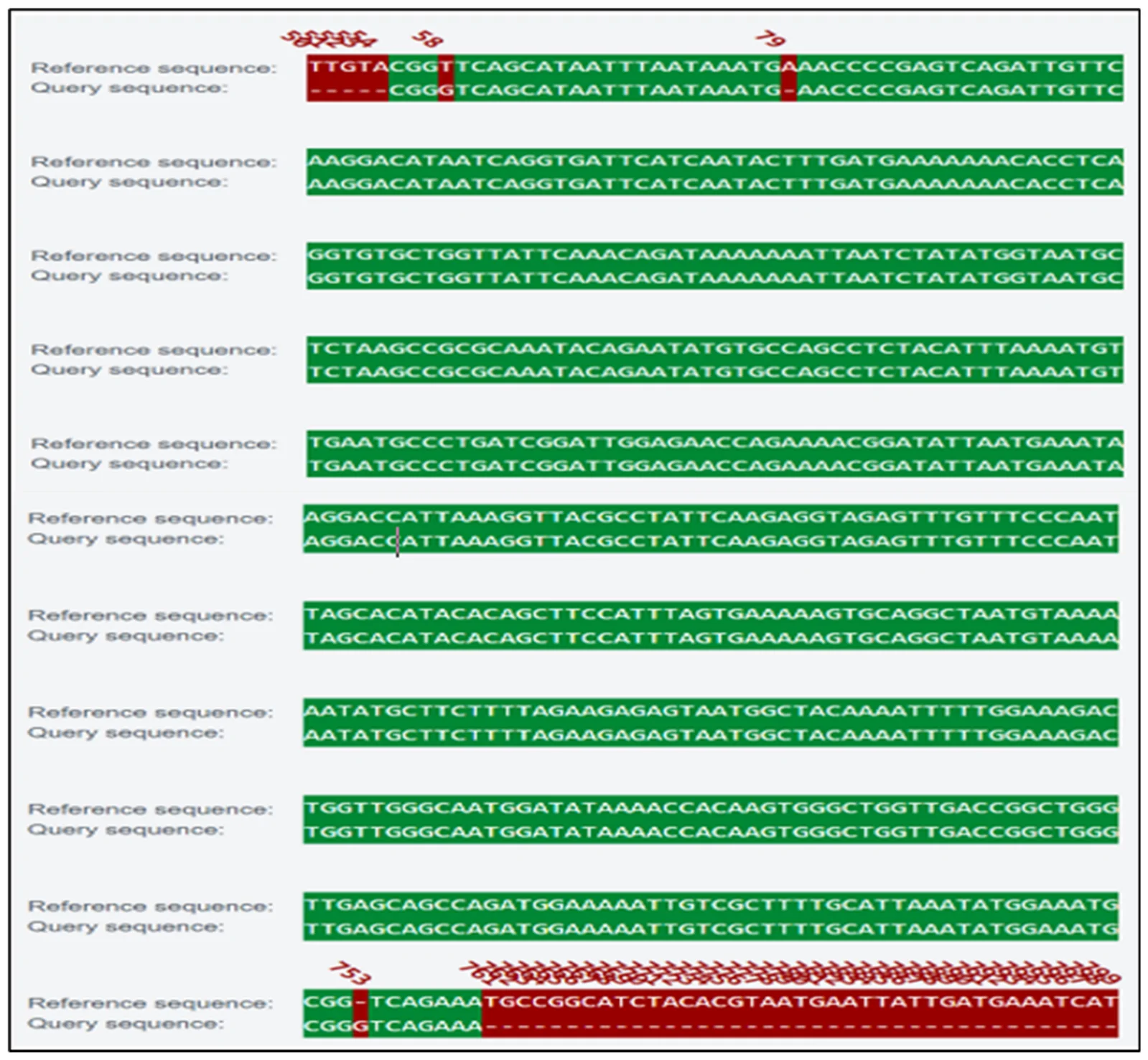
Nucleotide alignment of *blaOXA-23* and *blaNDM-1* sequences, showing high homology with global reference sequences. Where green shading indicates nucleotide positions identical between the query and reference sequences; red shading indicates mismatches, insertions, or deletions.

**Figure 6:**
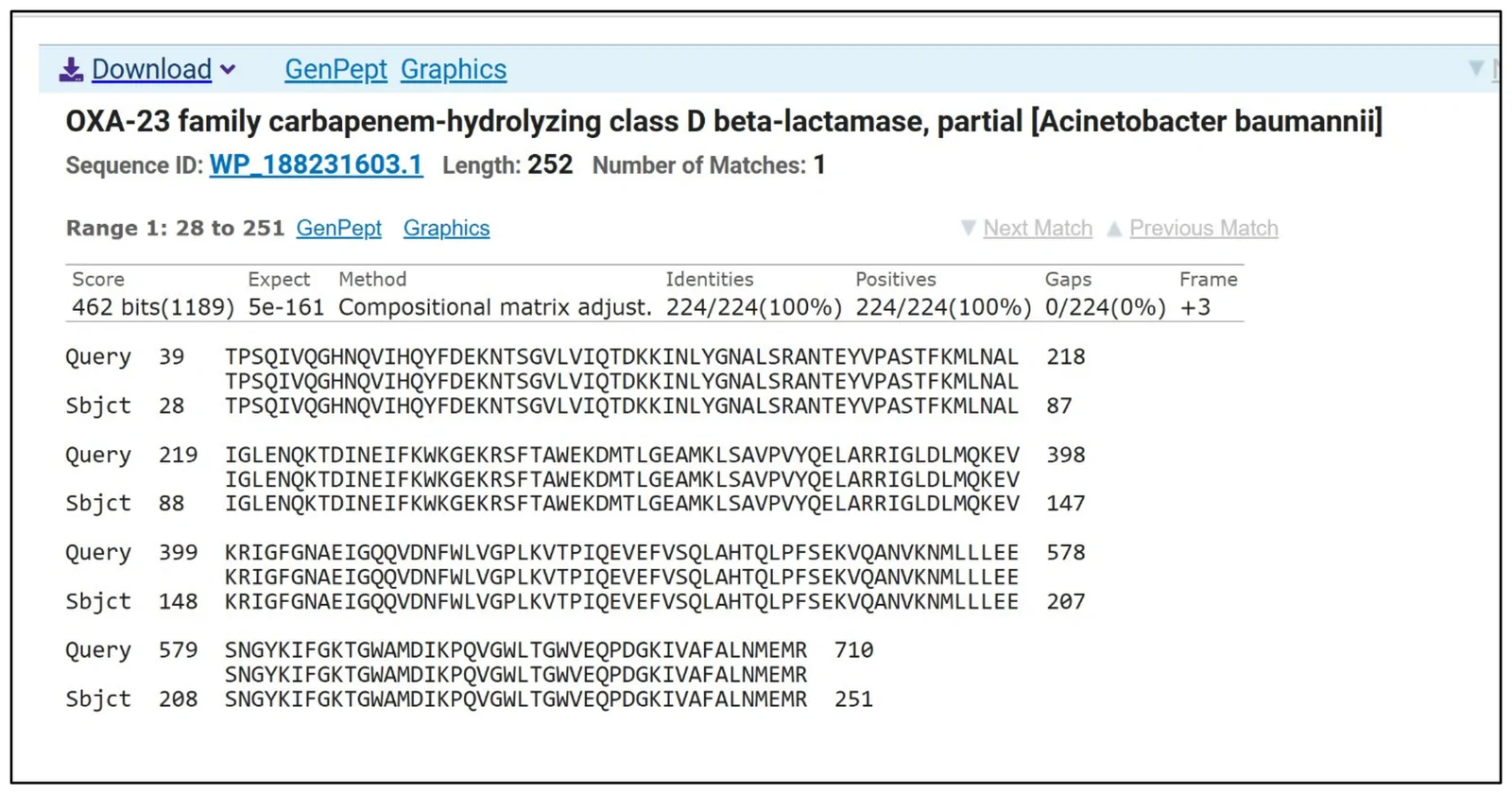
BLASTp alignment of the query protein sequence, showing 100% identity with the *OXA-23* family carbapenem-hydrolyzing class D β-lactamase from *A. baumannii*.

Sanger sequencing of the representative *blaOXA-23* and *blaNDM-1* amplicons followed by BLASTn analysis showed 98–100% nucleotide identity with corresponding reference sequences in the NCBI GenBank database (Figure 5). On review of the translated open reading frame, no frameshift mutations or premature stop codons were detected. The T-to-G substitution observed in *blaOXA-23* was synonymous, and the previously noted insertion/deletion signals corresponded to alignment gaps or terminal low-quality sequence positions that were not retained in the translated coding sequence. BLASTp analysis showed 100% predicted amino acid identity with the reference OXA-23 protein sequence (Figure 6). No nucleotide variation was observed in the analyzed *blaNDM-1* sequence. These findings may indicate a high sequence similarity of the detected genes.

## 3. Materials and Methods

### 3.1. Ethical Approval and Study Design

This study received ethical approval from the Research Ethics Committee of the Department of Microbiology, Kohat University of Science and Technology (Ref. No. KUST/Ethical Committee1624/). A cross-sectional study was conducted from November 2023 to February 2024 at Khalifa Gul Nawaz Teaching Hospital, Bannu, Khyber Pakhtunkhwa, Pakistan. Patients presenting clinical suspicion of healthcare-associated infections following general surgical procedures, including post-operative wound infections, diabetic foot infections, and hernia mesh site infections, were eligible for inclusion. Demographic data (age, sex) and relevant clinical information, including underlying comorbidities and surgical history, were recorded. Patients who had received systemic antibiotic therapy within 72 h prior to sample collection, as well as those with contaminated or unsuitable specimens, were excluded from the study. All specimens were collected under strictly aseptic conditions and transported immediately to the Department of Microbiology, KUST, where culture, phenotypic characterization, and molecular analysis were performed.

### 3.2. Sample Collection and Bacterial Culture

Surgical wound specimens (n = 118) were collected aseptically using sterile cotton swabs. Swabs were immediately placed in transport medium and transferred to the microbiology laboratory under cold chain conditions (4 °C) to minimize bacterial overgrowth during transit. Specimens were inoculated onto nutrient agar, MacConkey agar, and blood agar plates, and incubated aerobically at 37 °C for 24–48 h. Following incubation, morphologically distinct colonies were examined, and representative colonies were sub-cultured onto appropriate media to obtain pure bacterial isolates for further identification and antimicrobial susceptibility testing [10].

### 3.3. Bacterial Identification

*A. baumannii* isolates were provisionally identified based on their characteristic colony morphology (creamy, non-hemolytic, mucoid colonies on blood agar), Gram-negative coccobacillary appearance on microscopy, and standard biochemical test results, including catalase and urease reactions, as well as oxidase and indole tests. Molecular confirmation of species identity was achieved by PCR amplification and Sanger sequencing of the 16S rRNA gene, and the resulting sequences were compared against reference sequences in the NCBI GenBank database using BLASTn analysis, with identity thresholds of 98–100% accepted for species confirmation [11].

### 3.4. In Vitro Antimicrobial Susceptibility Testing

In vitro antimicrobial susceptibility testing was performed by the Kirby–Bauer disk diffusion method, in accordance with the Clinical and Laboratory Standards Institute [12]. Briefly, freshly grown bacterial colonies were suspended in sterile normal saline and adjusted to a 0.5 McFarland turbidity standard (approximately 1.5 × 10^8^ CFU/mL). The standardized inoculum was evenly spread onto Mueller–Hinton agar (MHA) plates using sterile cotton swabs. After allowing the agar surface to dry for 10–15 min, commercially available antimicrobial disks were placed aseptically. The following antibiotic disks were tested: imipenem (10 µg), meropenem (10 µg), ceftazidime (30 µg), cefepime (30 µg), cefotaxime (30 µg), levofloxacin (5 µg), ciprofloxacin (5 µg), ofloxacin (5 µg), gentamicin (10 µg), aztreonam (30 µg), polymyxin B (300 U), and colistin (10 µg). Plates were incubated aerobically at 35 °C for 18–24 h. Inhibition-zone diameters were measured in millimeters and interpreted as susceptible (S) or resistant (R) according to CLSI 2024 for *Acinetobacter* spp. Isolates resistant to at least one agent from three or more antibiotic classes were classified as multidrug-resistant (MDR) [13].

### 3.5. Concentration-Dependent Agar Well Diffusion Assay of Imipenem and Meropenem

Agar well diffusion was used to assess concentration-dependent inhibition-zone responses of imipenem and meropenem against MDR A. baumannii isolates [14]. Mueller–Hinton agar plates were inoculated with a standardized bacterial suspension adjusted to a 0.5 McFarland standard (approximately 1.5 × 10^8^ CFU/mL). Wells of 6 mm diameter were punched aseptically using a sterile cork borer. Serial concentrations of imipenem and meropenem were prepared in sterile distilled water at 10, 13, 15, 17, 19, 21, 23, 25, and 27 µg/mL, and 100 µL of each concentration was dispensed into the wells. Plates were incubated aerobically at 35 ± 2 °C for 18–20 h. Zones of inhibition were measured in millimeters from the edge of the well to the edge of the clear inhibition zone. Results were reported as inhibition-zone diameters at each tested concentration. The reported values represent single measurements obtained for each concentration.

### 3.6. Genomic DNA Extraction

The bacterial genomic DNA was extracted by following the phenol–chloroform–isoamyl alcohol (PCI) method [15]. Briefly, mid- to late-log-phase bacterial cultures were pelleted by centrifugation, resuspended in buffer containing RNase A, and treated with lysozyme, achromopeptidase, SDS, and proteinase K for cell lysis. DNA was purified by phenol–chloroform–isoamyl alcohol extraction, precipitated with ethanol, resuspended in TE buffer, and stored at 4 °C until PCR analysis. DNA quality was checked by electrophoresis on 1.5% agarose gel and visualization under a UV transilluminator.

### 3.7. Bacterial Strain Confirmation Using 16S rRNA Gene

Molecular identification of bacterial strains was carried out by using 16S rRNA gene amplification and universal primers [16], as given in Table 5. The PCR mixture (total volume 50 µL) contained 25 µL of 2× PCR master mix (Thermo-scientific, Waltham, MA, USA), 2 µL each of forward and reverse primers (10 pmol/µL), 5 µL of extracted DNA, and 16 µL of nuclease-free water. Thermal cycling conditions were as follows for 16S rRNA: initial denaturation at 95 °C for 5 min; 30 cycles of denaturation at 95 °C for 30 s, annealing at 55 °C for 30 s, and extension at 72 °C for 1 min; and a final extension at 72 °C for 5 min [17]. Then, after amplification, the PCR products were checked using 2% agarose gel, purified, and then sent for Sanger sequencing (Macrogene, Seoul, Republic of Korea) for further confirmation and to compare it with already reported cases.

### 3.8. Molecular Detection of Antibiotic Resistance Genes

The primers used for analysis of resistance genes were adopted from previous studies [7]. The PCR was performed in a total volume of 25 µL, consisting of 3 µL of template DNA from each isolate, 12.5 µL of green PCR master mix (Thermo), 0.5 µL of each primer, and PCR-grade water to complete the volume. The PCR products were electrophoresed on a 2% agarose gel and visualized under a UV transilluminator for confirmation and detection of resistance genes. A 100 bp DNA ladder was run alongside the samples to determine the size of the PCR amplicon [18]. Then, after amplification, the PCR products were checked using 2% agarose gel, purified, and then sent for Sanger sequencing (Macrogene, Republic of Korea) for further confirmation and to compare it with already reported cases.

### 3.9. Bioinformatics Analysis and Phylogenetic Tree Construction

Raw Sanger sequencing chromatograms for the 16S rRNA, *blaOXA-23*, and *blaNDM-1* gene amplicons were quality-checked and edited using Sequencing Analysis software (version 5.1.1). Validated sequences were assembled and subjected to BLASTn homology searches against the NCBI GenBank nucleotide database (http://blast.ncbi.nlm.nih.gov/Blast.cgi) (accessed on 16 June 2026) to confirm species identity and gene variant classification. The partial 16S rRNA gene sequences and representative *blaOXA-23* and *blaNDM-1* sequences of confirmed isolates were deposited in GenBank. Using the neighbor-joining method, a phylogenetic tree was constructed in Mega software (version 11). Bootstrap values were analyzed based on 1000 replications. Resistance genes (*blaOXA-23* and *blaNDM-1)* were additionally analyzed by BLASTp to assess sequence identity at the protein level, and to evaluate any potential effects of identified nucleotide substitutions on conserved carbapenemase motifs and enzyme structures.

## 4. Discussion

Carbapenem-resistant *Acinetobacter baumannii* (CRAB) has become one of the most clinically significant nosocomial pathogens in healthcare settings globally, with post-surgical patients representing a particularly vulnerable population due to disrupted physiological barriers and frequent exposure to broad-spectrum antimicrobials [19]. The present study investigated the prevalence of antimicrobial resistance profiles and selected carbapenemase genes of *A. baumannii* isolated from post-surgical patients at Khalifa Gul Nawaz Teaching Hospital, Bannu, Khyber Pakhtunkhwa, Pakistan, providing local baseline data from an underrepresented surgical setting.

In the present study, *A. baumannii* was isolated from 19.5% (23/118) of surgical wound specimens, with a higher number of positive isolates observed among male patients and in the 51–68-year age group. The most common associated clinical conditions were diabetic foot infections (47%), post-mesh hernia infections (34%), and post-appendectomy wound infections (17%). The predominance of diabetic foot infections is consistent with a previous Pakistani study reporting MDR Gram-negative pathogens, including *A. baumannii*, in diabetic foot infections and hospitalized patients [7]. Molecular identification by 16S rRNA gene sequencing confirmed 98 to 100% nucleotide identity with reference *A. baumannii* sequences in the NCBI GenBank for all 23 isolates. Phylogenetic analysis revealed that the study isolates clustered closely with geographically diverse reference strains, which is consistent with the conserved nature of the 16S rRNA gene and supports its reliability for species confirmation in resource-limited settings, as previously validated by [20].

Overall, 91.3% of isolates were MDR, with carbapenem resistance recorded in 82.6% for both imipenem and meropenem, complete resistance to aztreonam and gentamicin (100%), and fluoroquinolone resistance in 91.3% of isolates. In the local surgical setting, this resistance pattern may reflect antimicrobial selection pressure caused by frequent exposure to broad-spectrum antibiotics [21]. These findings also suggest that empirical use of carbapenems, fluoroquinolones, or aminoglycosides may be unreliable for suspected post-surgical *A. baumannii* infections and should be guided by local susceptibility data.

Colistin demonstrated the greatest retained activity, with 60.9% of isolates remaining susceptible. However, colistin resistance was detected in 39.1% of isolates, a clinically concerning finding given its role as a last-resort therapeutic agent. Previous studies have associated colistin resistance in *A. baumannii* with chromosomal mutations in the lpxA, lpxC, and lpxD genes, as well as in plasmid-mediated mcr determinants [22].

The concentration-dependent agar well diffusion assay provided additional phenotypic information on the response of MDR *A. baumannii* isolates to imipenem and meropenem. Across the tested concentration range of 10–27 µg/mL, both antibiotics showed a gradual increase in inhibition-zone diameters, indicating that stronger inhibition was observed only with increasing antibiotic exposure. This pattern supports the reduced carbapenem activity observed by standard disk diffusion. Similar findings have been reported in carbapenem-resistant Gram-negative bacteria, where resistant strains required higher antibiotic exposure to demonstrate measurable inhibition [23]. Molecular screening identified *blaOXA-23* in 60.9% and *blaNDM-1* in 30.4% of isolates, with co-expression of both genes in four isolates. In the present hospital setting, the predominance of *bla*OXA-23, together with high imipenem and meropenem resistance, suggests that this gene may be an important contributor to the local carbapenem-resistant phenotype. This may reflect local antimicrobial selection pressure from repeated or prolonged use of broad-spectrum beta-lactams, including carbapenems. The detection of *bla*NDM-1 is also clinically important, as NDM-type carbapenemases can further compromise beta-lactam treatment options [24]. Co-detection of both genes confers resistance to virtually all available beta-lactam antibiotics, representing a particularly challenging therapeutic scenario.

Sequence analysis of representative *blaOXA-23* and *blaNDM-1* amplicons confirmed 98 to 100% nucleotide identity with reference *blaOXA-23* and *blaNDM-1* sequences in the NCBI GenBank database. For *bla*OXA-23, nucleotide-level variations were observed in the analyzed sequence. Translation of the validated coding region and comparison with reference OXA-23 protein sequences showed no detectable amino-acid substitutions in the analyzed region. Similar sequence conservation of carbapenemase genes has been reported previously [25].

### Study Limitations

The present study has several limitations. First, this was a single-center study conducted over a limited period, and the number of *A. baumannii* isolates was small. Therefore, the findings should be interpreted as local baseline data and should not be generalized to the entire province or country. Second, species confirmation was based on phenotypic tests and 16S rRNA gene sequencing. Because members of the *Acinetobacter calcoaceticus–baumannii* complex are closely related, additional confirmatory methods, such as *rpoB* sequencing, gyrB analysis, MALDI-TOF MS, or whole-genome sequencing, would strengthen species identification. Third, molecular analysis was limited to *bla*OXA-23 and *bla*NDM-1, while other resistance mechanisms, including *bla*OXA-24/40, *bla*OXA-58, intrinsic *bla*OXA-51-like genes, ISAba1-associated overexpression, efflux pumps, porin alterations, and penicillin-binding protein modifications, were not investigated. Fourth, MLST, PFGE, cgMLST, and whole-genome sequencing were not performed, so clonal relatedness and transmission pathways could not be determined. Fifth, imipenem and meropenem were selected as clinically relevant carbapenems for *Acinetobacter* spp.; ertapenem was not included because it is not routinely used as the principal interpretive carbapenem for *Acinetobacter* spp. Sixth, colistin was included in the antimicrobial susceptibility panel because it remains a clinically important and locally available therapeutic option for multidrug-resistant and carbapenem-resistant *A. baumannii* infections; however, colistin susceptibility was assessed by disk diffusion, which is not recommended for definitive colistin testing. Finally, the agar well diffusion assay provided concentration-dependent inhibition-zone data and should not be interpreted as a MIC determination.

## 5. Conclusions

The present study provides local baseline data on multidrug-resistant and carbapenem-resistant *Acinetobacter baumannii* isolated from post-surgical infection cases at Khalifa Gul Nawaz Teaching Hospital, Bannu, Khyber Pakhtunkhwa, Pakistan. A high proportion of isolates showed resistance to multiple antibiotic classes, including imipenem, meropenem, fluoroquinolones, aztreonam, and gentamicin, suggesting limited treatment options in this setting. Molecular screening detected *bla*OXA-23 more frequently than *bla*NDM-1, with co-detection of both genes in some isolates. These findings suggest that selected carbapenemase genes may contribute to carbapenem resistance among the study isolates.

## 6. Recommendations

Future studies should include a larger number of isolates from multiple hospitals to better understand the distribution of carbapenem-resistant *A. baumannii* in post-surgical infections. Additional confirmatory methods, such as *rpoB* sequencing, MALDI-TOF MS, or whole-genome sequencing, may strengthen species identification and molecular characterization. Standardized MIC-based methods, particularly for carbapenems and colistin, should be used in future antimicrobial susceptibility testing. Broader gene resistance analysis and molecular typing may also help clarify resistance mechanisms and possible transmission patterns. At the hospital level, regular antibiogram monitoring, antimicrobial stewardship, hand hygiene, environmental cleaning, and infection-control practices should be strengthened in surgical units.

## Figures and Tables

**Table 1:** Demographic and clinical distribution of *A. baumannii*-positive cases among surgical site infection (SSI) patients (n = 23) at Khalifa Gul Nawaz Teaching Hospital, Bannu, Khyber Pakhtunkhwa, Pakistan (November 2023 to February 2024).

Distribution	Number of Positive Cases
Aged 10 to 22	1
Aged 24 to 50	6
Aged 51 to 68	16
**Gender**	
Female	7
Male	16
**Associated diseases**	
Diabetic patients	11
Post-mesh hernia	8
Appendectomy	4

**Table 2:** In vitro antimicrobial resistance profiles of *Acinetobacter baumannii* isolates (n = 23) determined by Kirby–Bauer disk diffusion method and interpreted according to CLSI 2024 breakpoints for *Acinetobacter* spp., where R—resistant; I—intermediate; and S—susceptible.

Antibiotic	Resistant Isolates n (%)	Intermediate Isolates n (%)	Susceptible Isolates n (%)
**Imipenem**	19 (82.6%)	1 (4.3%)	3 (13.0%)
**Meropenem**	19 (82.6%)	1 (4.3%)	3 (13.0%)
**Levofloxacin**	21 (91.3%)	0 (0.0%)	2 (8.6%)
**Ciprofloxacin**	21 (91.3%)	1 (4.3%)	1 (4.3%)
**Ofloxacin**	21 (91.3%)	1 (4.3%)	1 (4.3%)
**Aztreonam**	23 (100%)	0 (0.0%)	0 (0.0%)
**Gentamicin**	23 (100%)	0 (0.0%)	0 (0.0%)
**Polymyxin B**	16 (69.5%)	0 (0.0%)	7 (30.5%)
**Colistin**	9 (39.1%)	0 (0.0%)	14 (60.9%)

**Table 3:** Concentration-dependent zones of inhibition (mm) of imipenem and meropenem against multidrug-resistant *Acinetobacter baumannii* isolates (n = 21), as determined by agar well diffusion at concentrations ranging from 10 to 27 µg/mL. Inhibition zones were interpreted in mm.

Concentration (µg/mL)	Imipenem ZOI (mm)	Meropenem ZOI (mm)
**10**	0	7
**13**	0	10
**15**	8	13
**17**	11	15
**19**	14	16
**21**	17	17
**23**	20	19
**25**	23	22
**27**	25	25

**Table 4:** Antibiotic resistance according to the detection of *bla*OXA-23 and *bla*NDM-1 carbapenemase genes in selected multidrug-resistant *A. baumannii* isolates.

Antibiotic	Resistance Among *bla*OXA-23-Positive Isolates N = 14	Resistance Among *bla*NDM-1-Positive Isolates N = 7	Resistance Among Selected MDR Isolates N = 21
Imipenem	9/14 (64.3%)	5/7 (71.4%)	13/21 (61.9%)
Meropenem	9/14 (64.3%)	5/7 (71.4%)	13/21 (61.9%)
Polymyxin B	9/14 (64.3%)	5/7 (71.4%)	16/21 (76.2%)
Levofloxacin	9/14 (64.3%)	7/7 (100%)	15/21 (71.4%)
Ofloxacin	9/14 (64.3%)	5/7 (71.4%)	13/21 (61.9%)
Gentamicin	9/14 (64.3%)	5/7 (71.4%)	13/21 (61.9%)
Tetracycline	0/14 (0.0%)	0/7 (0.0%)	0/21 (0.0%)
Piperacillin/Tazobactam	9/14 (64.3%)	7/7 (100%)	15/21 (71.4%)
Aztreonam	11/14 (78.6%)	7/7 (100%)	17/21 (81.0%)
Colistin	6/14 (42.9%)	5/7 (71.4%)	10/21 (47.6%)

**Table 5:** Nucleotide sequences of primers used for PCR amplification of the 16S rRNA, *blaOXA-23*, and *blaNDM-1* genes in the present study.

S.No	Genes	Sequences of Primers	Tm	Product Size	Reference
(1)	*blaNDM-1*	F-5′-CCAATATTATGCACCCGGTCG-3′ R-5′-ATGCGGGCCGTATGAGTGATTG-3′	58.85 59.07	850 bp	[7]
(2)	*blaOXA-23*	F-′5′-GATGTGTCATAGTATTCGTCGT-3′ R-5′-TCACAACAACTAAAAGCACTGT-3′	58.0 57.0	742 bp	
(3)	16s rRNA	F-5′-AGAGTTTGATYMTGGCTCAG-3′ R-5′-AGAAAGGAGGTGATCCAGCC-3′	57.6 58.5	1480 bp	[16]

## Data Availability

The data presented in this study are not publicly available due to privacy and ethical restrictions, as the data involve clinical samples collected from post-surgical patients. The data may be made available by the corresponding author upon reasonable request, subject to institutional and ethical approval.
